# Translational studies of intravenous and intracerebroventricular routes of administration for CNS cellular biodistribution for BMN 250, an enzyme replacement therapy for the treatment of Sanfilippo type B

**DOI:** 10.1007/s13346-019-00683-6

**Published:** 2020-01-15

**Authors:** Anita Grover, Danielle Crippen-Harmon, Lacey Nave, Jon Vincelette, Jill C. M. Wait, Andrew C. Melton, Roger Lawrence, Jillian R. Brown, Katherine A. Webster, Bryan K. Yip, Brian Baridon, Catherine Vitelli, Sara Rigney, Terri M. Christianson, Pascale M. N. Tiger, Melanie J. Lo, John Holtzinger, Adam J. Shaywitz, Brett E. Crawford, Paul A. Fitzpatrick, Jonathan H. LeBowitz, Sherry Bullens, Mika Aoyagi-Scharber, Stuart Bunting, Charles A. O’Neill, Jason Pinkstaff, Anil Bagri

**Affiliations:** BioMarin Pharmaceutical Inc., 105 Digital Drive, Novato, CA 94949 USA

**Keywords:** Lysosomal storage diseases, Enzyme replacement therapy, Sanfilippo type B, Alpha-*N*-acetylglucosaminidase, Mucopolysaccharidosis type III, Intracerebroventricular delivery

## Abstract

BMN 250 is being developed as enzyme replacement therapy for Sanfilippo type B, a primarily neurological rare disease, in which patients have deficient lysosomal alpha-*N*-acetylglucosaminidase (NAGLU) enzyme activity. BMN 250 is taken up in target cells by the cation-independent mannose 6-phosphate receptor (CI-MPR, insulin-like growth factor 2 receptor), which then facilitates transit to the lysosome. BMN 250 is dosed directly into the central nervous system via the intracerebroventricular (ICV) route, and the objective of this work was to compare systemic intravenous (IV) and ICV delivery of BMN 250 to confirm the value of ICV dosing. We first assess the ability of enzyme to cross a potentially compromised blood–brain barrier in the *Naglu*^−/−^ mouse model and then assess the potential for CI-MPR to be employed for receptor-mediated transport across the blood–brain barrier. In wild-type and *Naglu*^−/−^ mice, CI-MPR expression in brain vasculature is high during the neonatal period but virtually absent by adolescence. In contrast, CI-MPR remains expressed through adolescence in non-affected non-human primate and human brain vasculature. Combined results from IV administration of BMN 250 in *Naglu*^−/−^ mice and IV and ICV administration in healthy juvenile non-human primates suggest a limitation to therapeutic benefit from IV administration because enzyme distribution is restricted to brain vascular endothelial cells: enzyme does not reach target neuronal cells following IV administration, and pharmacological response following IV administration is likely restricted to clearance of substrate in endothelial cells. In contrast, ICV administration enables central nervous system enzyme replacement with biodistribution to target cells.

## Introduction

BMN 250 is being developed as an intracerebroventricular (ICV) delivered enzyme replacement therapy (ERT) for the treatment of Sanfilippo syndrome type B (mucopolysaccharidosis IIIB), which results from deficiency in activity of the enzyme alpha-*N*-acetylglucosaminidase (NAGLU). NAGLU is one of the enzymes required for the degradation of heparan sulfate (HS) glycosaminoglycans in the lysosome. When NAGLU activity is deficient, specific HS fragments cannot be processed and accumulate throughout the body. In contrast to many lysosomal storage diseases, Sanfilippo patients exhibit a predominantly neurological phenotype, and, given this disease pathophysiology, successful treatment is believed to require replacement of enzyme activity directly within the central nervous system (CNS) due to protection of the CNS by the blood–brain barrier (BBB).

BMN 250 is a fusion protein comprised of recombinant human NAGLU fused with truncated human insulin-like growth factor 2 (IGF2). BMN 250 is enzymatically active and taken up by cells via the cation-independent mannose 6-phosphate receptor (CI-MPR), also known as IGF2 receptor (IGF2R) [1]. Mannose 6-phosphate (M6P) glycosylation generally targets enzymes to be trafficked to the lysosome. The IGF2 “tag” on BMN 250 affords glycosylation-independent lysosomal targeting via binding of the tag to CI-MPR/IGF2R, allowing transit of BMN 250 directly to the lysosome.

The *Naglu*^−/−^ mouse is a model of Sanfilippo type B [2] that recapitulates many biochemical aspects of the human disease, such as HS accumulation. This mouse model also shows marked CNS pathology, such as increased expression of markers of lysosomal pathophysiology, including greater than 2-fold elevated β-hexosaminidase (β-hex) activity and 3- to 10-fold elevated lysosome-associated membrane protein 2 (LAMP2) levels [1]. Importantly, neurons show particular pathology in *Naglu*^−/−^ mice [3, 4]. ICV delivery of BMN 250 directly into the ventricular cerebrospinal fluid (CSF) space normalized HS and the disease-specific non-reducing end (NRE) fragment levels and neuropathology observed in the *Naglu*^−/−^ mouse [1]. While ICV delivery may be considered invasive in comparison to systemic delivery, direct administration into the ventricular space circumvents the BBB.

The BBB is the primary barrier and regulatory interface between the intravascular space and brain parenchyma. It is composed of capillary endothelial cells, astrocyte end-feet, capillary basement membrane, and pericytes within the basement membrane [5]. Receptor-mediated pathways exist for transit across the BBB, including one proposed to be mediated by CI-MPR [6–8].

CI-MPR has been indirectly shown to facilitate trans-BBB transport of β-glucoronidase and sulfamidase in neonatal, but not adult wild-type (WT) mice, in a M6P-dependent manner [7, 8]. Similarly, a recombinant human NAGLU with enhanced mannose 6-phosphorylation has been proposed to provide sufficient brain distribution for therapeutic benefit when dosed systemically [6]. It has also been proposed that compromised BBB in *Naglu*^−/−^ mice resulting from disease-specific pathology may allow for brain distribution by systemically delivered of ERT [9].

Thus, the objective of this work was to compare systemic intravenous (IV) and ICV delivery of BMN 250 to confirm the value of ICV dosing. CI-MPR expression across developmental ages in both brain parenchyma and vasculature was assessed in WT and *Naglu*^−/−^ mouse, non-human primate (NHP), and human brain samples in order to define the age dependence and translatability of findings. Within the mouse disease model, we assessed the ability of diseased BBB to allow biodistribution and pharmacological activity of the enzyme in the CNS. Finally, the ability of CI-MPR to transport enzyme was studied in NHPs dosed via IV and ICV using biodistribution and IHC-based cellular localization. Collectively, these studies interrogate BBB integrity and receptor-mediated transport via CI-MPR as proposed mechanisms for trans-BBB penetration of ERT and show that neither mechanism is sufficient to convey meaningful distribution to the CNS following systemic administration and support the ICV administration route as an important advancement for addressing neurological disease.

## Materials and methods

### Animal studies

All study protocols were approved by an Institutional Animal Care and Use Committee.

### Developmental time course in mice

*Naglu*^−/−^ transgenic mice [2] (*Naglu*^−/−^, B6.129S6-*Naglu*^*tm1Efn*^/J, The Jackson Laboratory, 00827, *n* = 5/age group) and WT controls (*Naglu*^+/+^, *C57BL/6J*, The Jackson Laboratory, 00664, *n* = 4–5/age group) were enrolled and euthanized at various ages spanning p3 to ≥ 12 weeks. Tissues were fixed in situ with 10% neutral buffered formalin for immunohistochemistry (IHC) analysis.

### Systemic treatment in 12-week-old mice

**Fig. 1:**
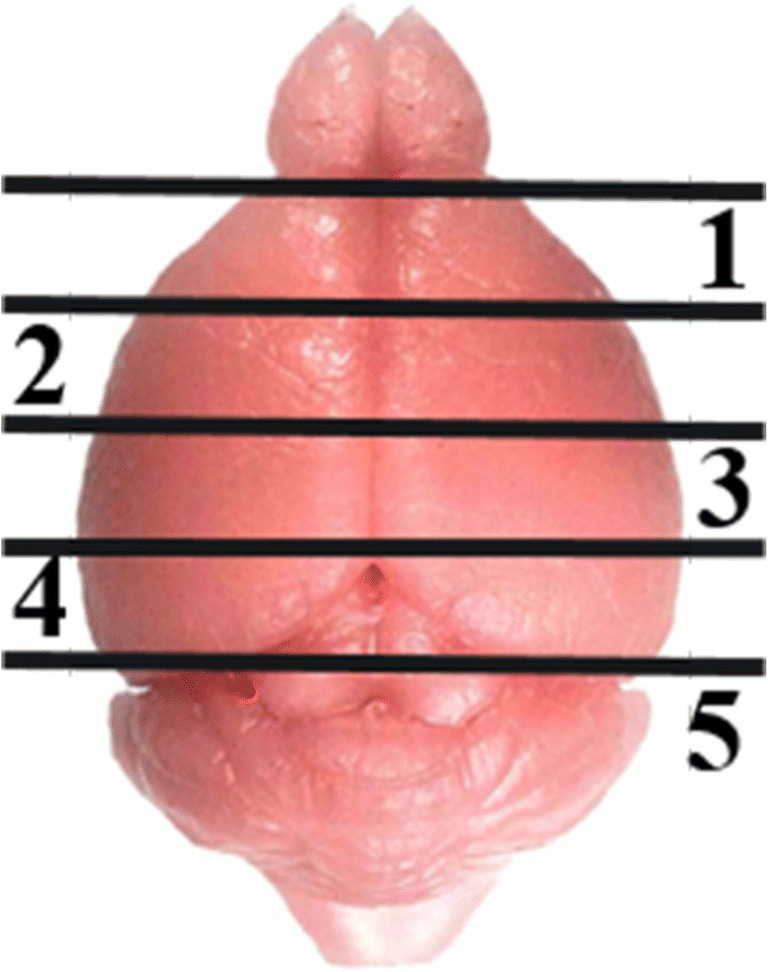
Mouse brain slices

*Naglu*^−/−^ transgenic mice [2] (*Naglu*^−/−^, B6.129S6-*Naglu*^*tm1Efn*^/J, The Jackson Laboratory, 00827) received either BMN 250 or vehicle twice weekly for 2 weeks via tail vein IV infusion. All mice, *n* = 4 male and *n* = 4 female per group (*n* = 16 mice total), were approximately 12 weeks of age at study start. Groups were randomized with respect to body weight and sex, although in previous studies no sex difference has been observed in the brain (data not shown). Mice were euthanized 1 day following the final infusion, and the brain was harvested into slices as indicated in Fig. 1. Brain slices 1 and 3 were processed for detection of NAGLU, HS and NRE, and β-hex. Brain slices 2 and 4 were fixed in 10% neutral buffered formalin processed for detection of NAGLU and LAMP2 via IHC.

### NAGLU and LAMP2 IHC and image analysis in mice

NAGLU was assessed by staining with a NAGLU antibody shown previously to specifically detect rhNAGLU in *Naglu*^−/−^ mouse brains after ICV administration [1]. Lysosomal pathology was assessed by staining with a LAMP2 antibody also previously described [1].

### Enzyme and activity assays in mouse studies

Recombinant human NAGLU-IGF2 fusion protein (BMN 250) was expressed in Chinese hamster ovary cells and purified, as described elsewhere [1]. The purified protein (20 mg/mL) was stored frozen at − 80 °C in artificial CSF solution (Vehicle): 1 mM Na_2_HPO_4_/NaH_2_PO_4_, 148 mM NaCl, 3 mM KCl, 0.8 mM MgCl_2_, 1.4 mM CaCl_2_, pH 7.2. Prior to IV infusion, the enzyme was diluted, where necessary, to maintain the equivalent dosing volume throughout the studies. NAGLU enzyme activity in brain homogenate was determined using a synthetic fluorogenic substrate, 4-methylumbelliferyl (4MU)-*N*-acetyl-α-glucosaminide (EMD Millipore, Billerica, MA or Toronto Research Chemicals, North York, Canada), following a published protocol [5, 10] with minor modifications. β-hex activity was similarly determined using 4MU-*N*-acetyl-β-glucosaminide (EMD Millipore, Billerica, MA), as described previously. Activity is expressed as nanomoles of 4MU released per hour per total protein, which was estimated by Bradford assay with bovine serum albumin as standard.

### Quantification of total and disease-specific heparan sulfate in mouse brain

Total HS and the disease-specific NRE of HS in homogenized brain samples were quantified using the Sensi-Pro assay, following previously published procedures [11, 12] and as previously described [1]. Briefly, brain HS glycosaminoglycans were purified by anion exchange chromatography and digested with heparan lyases (IBEX Technologies). The depolymerization products were tagged and quantified by liquid chromatography-mass spectrometry. The measured quantity of total HS (internal disaccharides) and disease-specific NREs (trisaccharides, glucosamine-N-acetate alpha1,4 iduronate-2-sulfate alpha1,4 glucosamine 2-N-sulfate), referred to as A0I2S0 [13] was expressed as picomoles per milligram of brain wet weight.

### Measurement of vascular astrocytosis in mice

Formalin-fixed paraffin-embedded (FFPE) mouse brain sections were immunostained with goat anti-CD31 (AF3628, R&D Systems) alone or in co-stain with rabbit anti-GFAP (G9629, Sigma). Primary antibodies were detected using anti-goat immunoglobulin G (IgG) H&L (Alexa Fluor® 488) (A-11055, ThermoFisher Scientific) and anti-rabbit IgG H&L (Alexa Fluor® 555) (A-31573, ThermoFisher Scientific). For quantitative image analysis, whole mouse brain sections were scanned using a Leica Ariol slide scanning microscope with an HC PL APO 20×/0.7 objective and regions were extracted from every sample, one each from the cingulate cortex (cortical) and caudate putamen (subcortical). Using custom macros in ImageJ [14], the area of GFAP staining within a 3-μm distance from CD31-positive vessels was quantified to determine vascular astrocytosis. Statistics were analyzed in GraphPad Prism 6.

### Cynomolgus monkey tissue samples

Frozen brain tissue from two healthy, untreated, male cynomolgus monkeys aged 3.9 years was acquired from Northern BioMedical Research (Norton Shores, MI). Cores were cut from superficial cerebral cortex, cerebral cortex, striatum, thalamus, midbrain, occipital cortex, medulla, and cerebellum and transferred to pre-chilled 4% paraformaldehyde in phosphate-buffered saline and fixed for 48 h at 4 °C. Fixed cores were processed and embedded in paraffin. Tissues were co-immunostained for rabbit anti-CI-MPR (ab124767, Abcam) with either goat anti-CD31 (AF3628, R&D Systems) or mouse anti-NeuN (MAB377, Millipore). Primary antibodies were detected using anti-rabbit IgG H&L (Alexa Fluor® 488) (A-21206, ThermoFisher Scientific), anti-goat IgG H&L (Alexa Fluor® 555) (A-21432, ThermoFisher Scientific), or anti-mouse IgG H&L (Alexa Fluor® 555) (A-31570, ThermoFisher Scientific). For quantitative image analysis, whole mouse brain sections were scanned using a Leica Ariol slide scanning microscope with an HC PL APO 20×/0.7 objective and two regions were extracted from every sample, one each from the cingulate cortex and somatosensory cortex. Using Perkin Elmer Volocity (6.3), the percent of CD31-positive vessels that were also positive for CI-MPR was quantified. Statistics were analyzed in GraphPad Prism 6. Representative confocal images were acquired using a Leica TCS SP8 microscope with an HC PL APO 40×/1.30 or 63×/1.4 oil objective and 1-AU pinhole diameter.

### Human tissue samples

FFPE human cortical tissue from healthy donors was acquired from the NIH Neurobiobank at the University of Maryland, Baltimore, MD, for a range of ages: 2–3 months (*n* = 3), 1 year (*n* = 3), 2 years (*n* = 2), 3–7 years (*n* = 3), and 14–15 years (*n* = 3). FFPE human cortical tissue was immunostained with rabbit anti-CI-MPR (ab124767, Abcam) and detected using Impress anti-Rabbit HRP-conjugated secondary antibody (MP-7401, Vector Laboratories) followed by DAB substrate solution (SK-4100, Vector Laboratories). Images were acquired using a Leica DM5000 light microscope with 40× 0.85NA HC Plan Apo and 100× 1.4NA HCX Plan Apo objectives. DFC 550 top-mount camera and Leica LASX software were used.

### IV and ICV treatment in cynomolgus monkey

Healthy male juvenile cynomolgus monkeys 11–13 months of age and weighing approximately 1.4–1.9 kg were put on study (*n* = 7). Animals receiving ICV treatment (*n* = 5) were surgically implanted with ICV catheters in the left lateral ventricle for dose administration, and all animals were surgically implanted with intrathecal catheters in the lumbar spine for CSF sample collection. ICV and IV administration routes were approved under separate protocols; the protocols for both studies received approval by the Institutional Animal Care and Use Committee, and both studies were conducted in accordance with the United States Public Health Service’s Policy on Humane Care and Use of Laboratory Animals.

#### Drug administration to NHP

Animals were administered a single dose of vehicle or BMN 250. For animals receiving ICV treatment, approximately 2.5 mL of CSF was withdrawn via cisterna magna spinal tap for isovolumetric administration to minimize potential intracranial pressure changes. Animals receiving ICV administration were administered a single dose of vehicle (*n* = 2) or 73 mg (*n* = 3), the maximum feasible for ICV administration based on infusion volumes and drug concentration, of BMN 250 with an infusion rate of 0.5 mL/min for ~ 5 min. Animals receiving IV administration (*n* = 2) were administered a single IV dose of 200 mg/kg for a total approximate dose of 350 mg, the maximum feasible, of BMN 250 at a dose volume of 10 mL/kg and a rate of 3 mL/min. The maximum feasible dose was selected for the IV route to maximize the chance of detecting drug exposure in the CSF and CNS tissue.

#### CSF drug concentration in NHP

For ICV administered animals, pharmacokinetic samples were obtained from CSF from the lumbar spine at concentrations putatively close to the maximum (at the end of infusion and 0.5 h post-dose). For IV animals, CSF and plasma samples were collected and tested immediately prior to infusion and at 0.25, 1, 3, 6, 12, 24, 36, and 48 h post-dose. Samples were analyzed for BMN 250 concentration by electrochemiluminescent assay (ECLA), utilizing a biotinylated murine anti-IGF2 monoclonal capture antibody and ruthenylated goat anti-NAGLU polyclonal detection antibody in a sandwich format to detect BMN 250. The standard curve was generated using a 4-parameter logistic regression model. BMN 250 concentration in each sample was determined by interpolation from the standard calibrator curve and adjustment for sample dilution. The quantitative range for CSF and plasma assays was 8.23–2000 ng/mL.

#### CNS tissue biodistribution

At 48 h following dosing, animals were euthanized and specific tissues of the CNS were harvested and perfused. The 48-h time point for euthanasia was selected as the putative tissue *C*_max_. For determination of NAGLU tissue concentration, specimens of superficial and deep tissue, relative to the ventricle, from seven brain regions (cerebellum, cerebral cortex, medulla oblongata, midbrain, occipital cortex, striatum, and thalamus) and from three spinal cord regions (cervical, lumbar, and thoracic) were weighed and homogenized using a Precellys 24-Dual (Bertin Instruments, Montigny-le-Bretonneux, France). Tissues were analyzed for NAGLU concentration by ECLA and normalized to total protein. The IGF2 tag of BMN 250 is cleaved in lysosomes upon cellular uptake, and this tag was utilized to remove intact extracellular BMN 250 from samples using anti-IGF2 coupled magnetic beads. Quantitation of NAGLU in cynomolgus monkey brain and spinal cord tissue homogenates was performed using a sandwich ECLA with a rabbit anti-NAGLU polyclonal capture antibody and a ruthenylated goat anti-NAGLU polyclonal detection antibody. The standard curve was analyzed using a 4-parameter logistic regression model and the concentration of NAGLU in each sample was determined by interpolation from the standard calibrator curve and adjusted for sample dilution. The quantitative range was 4.92–200.0 ng/mL.

Because the doses administered ICV and IV were not the same, analyses are presented as dose-normalized: bioanalytical assay results were divided by total administered dose, such that comparisons were made on a per unit basis. Previous investigations (data not shown) with ICV and IV-administered BMN 250 to cynomolgus monkeys have shown dose proportionality following single and multiple doses in tissue, CSF, and plasma across the dose range studied here. Dose proportionality suggests dose-normalization is appropriate.

### Cynomolgus monkey IHC and image analysis

Cores (54 mm) were cut from frozen brain slices of selected brain regions with the highest NAGLU concentration following IV administration (superficial medulla oblongata and superficial midbrain). These regions were selected to maximize the likelihood of detecting NAGLU across cell types. Cores were transferred to pre-chilled 4% paraformaldehyde in phosphate-buffered saline and fixed for 48 h at 40 °C. Fixed cores were processed for FFPE using the Leica TP1020 and sectioned into 7 μm sections onto charged slides. Sections were deparaffinized and rehydrated to water, incubated in 10 μg/mL proteinase K in Tris-EDTA at 37 °C for 20 min and on benchtop for 10 min, and blocked in 2% normal donkey serum, 0.1% bovine serum albumin, 0.3% triton in tris-buffered saline for 1 h at room temperature. Sections were co-stained using a rabbit polyclonal antibody against BMN 250 (made in house, 1:400) and CD31 (ab9498, 1:100) and incubated overnight at 40 °C. Primary antibodies were detected with anti-rabbit IgG (H + L) Alexa Fluor® 488 (A21206, ThermoFisher Scientific) and anti-mouse IgG (H + L) Alexa Fluor® 555 (A31570, ThermoFisher Scientific). Sections were mounted in Fluoromount-G with DAPI (17984-24). Images were acquired on a Leica SP8 confocal microscope and adjusted in Adobe Photoshop using identical parameters for all samples.

## Results

### CI-MPR is developmentally regulated in brain vasculature, but not neurons, in wild-type mice

**Fig. 2:**
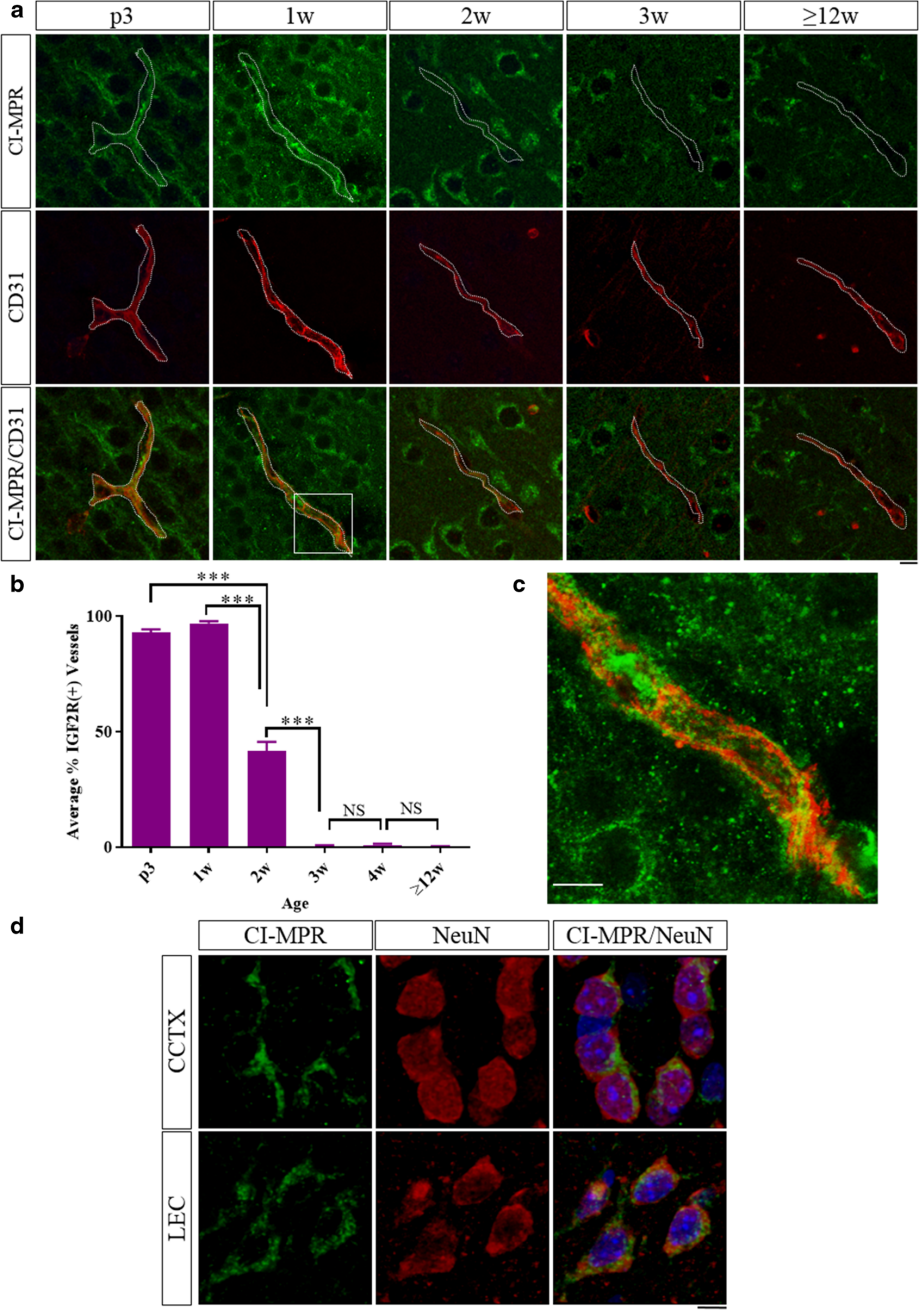
Brains of wild-type mice at different ages were co-stained for CI-MPR (green) and CD31 (red) (**a**, single-slice confocal images) and the percent of vessels positive for CI-MPR signal was quantified in the cortex (**b**). Single-slice high-resolution confocal image boxed region in **a** shows CI-MPR in both on the luminal and abluminal spaces of the CD31+ vessel (**c**). NeuN+ neuron (red) in cingulate and entorhinal cortices are positive for CI-MPR (green) at ≥ 12 weeks of age (**d**). ***p < 0.001, one-way ANOVA with Tukey’s multiple comparison’s test, scale bars = 10 μm (dotted line shows boundary of CD31 staining), error bars = SEM

Brain sections from WT mice at various ages from neonate to adult were co-stained by IHC for CI-MPR and CD31, a marker of endothelial cells (Fig. 2a) to quantify the CI-MPR signal associated with vasculature in cortical brain regions (Fig. 2b). At p3 and 1 week post-natal ages, nearly all the vessels in the cortex were positive for CI-MPR (93.0 ± 1.2% and 96.9 ± 0.9%, respectively). High-resolution confocal microscopy imaging shows the CI-MPR signal was qualitatively observed in the vessels both on the luminal and abluminal sides of the EC (Fig. 2c). By 2 weeks of age, less than 50% of the vessels in the cortex were positive for CI-MPR (41.4 ± 4.1%). At 3, 4, and ≥ 12 weeks of age, less than 1% of the vessels in the cortex were positive for CI-MPR signal (3 weeks: 0.49 ± 0.28%, 4 weeks: 0.98 ± 0.46%, and ≥ 12 weeks: 0.22 ± 0.16%). This trend was qualitatively observed throughout the brain in cortical regions as well as hippocampus, striatum, and thalamus, indicating the observed time course of CI-MPR expression in brain vasculature was a general trend and not region-specific.

CI-MPR was co-stained with the neuronal marker NeuN in adult (≥ 12 week old) mouse brains. In contrast to the vascular CI-MPR signal at this age, neurons in cingulate and lateral entorhinal cortices retain CI-MPR staining into adulthood (Fig. 2d), albeit with a qualitative decrease in staining intensity. Neuronal CI-MPR expression in adult tissue has been described previously [15, 16] and serves as an internal control for the vascular staining.

These data demonstrate that CI-MPR expression in endothelial cells of the WT mouse brain is developmentally regulated and declines precipitously over the first three weeks of post-natal life, while the same precipitous decline is not evident in neurons.

### CI-MPR regulation in brain vasculature and neurons are unchanged in *Naglu*^−/−^ mice compared to wild-type

**Fig. 3:**
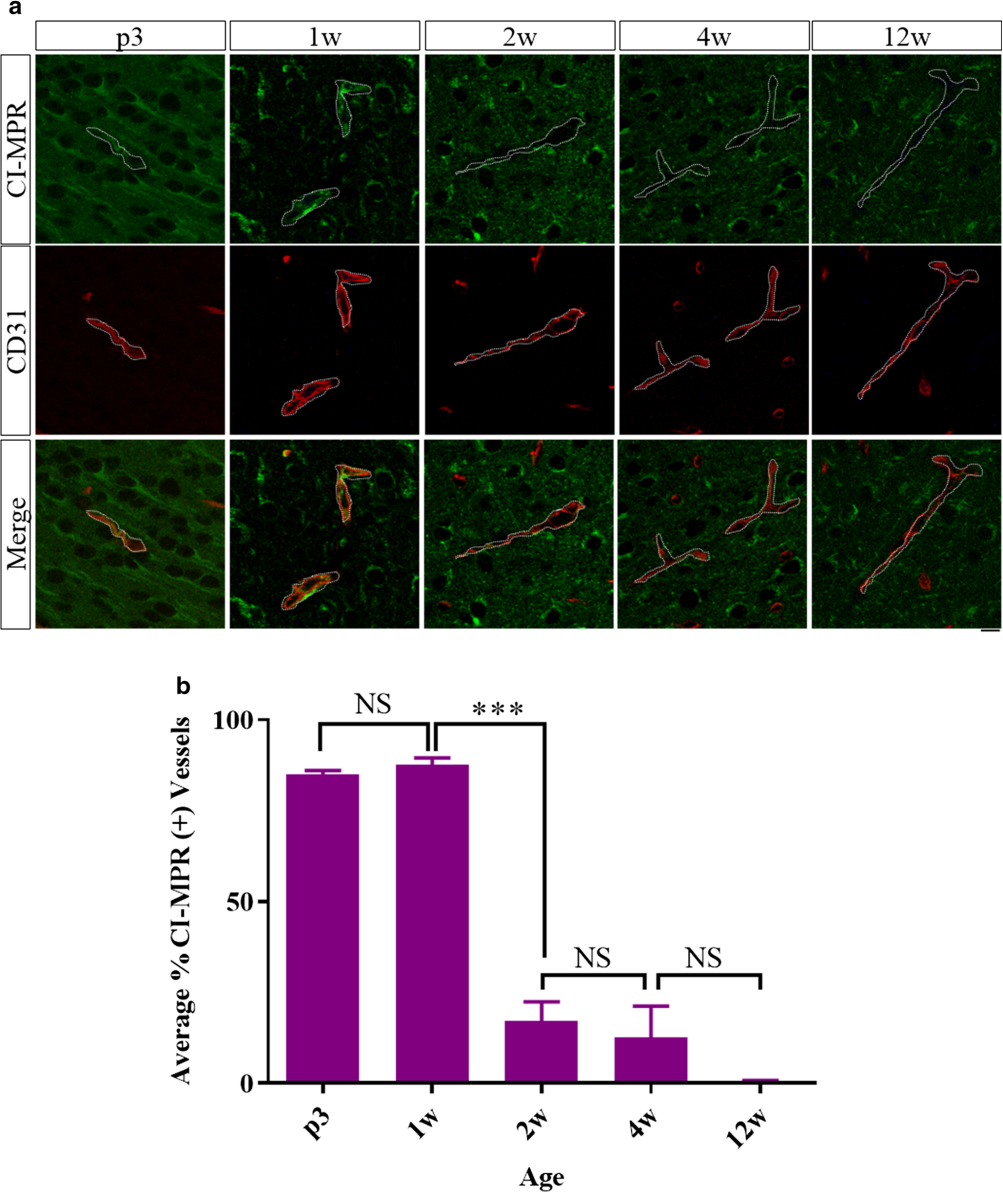
Brains of *Naglu*^−/−^ mice at different ages were co-stained for CI-MPR (green) and CD31 (red) (**a**, single-slice confocal images) and the percent of vessels positive for CI-MPR signal was quantified in the cortex (**b**). ***p < 0.001, one-way ANOVA with Tukey’s multiple comparison’s test, scale bar = 10 μm (dotted line shows boundary of CD31 staining), error bars = SEM

CI-MPR expression was evaluated in the *Naglu*^−/−^ mouse model to assess potential disease effects. Brains of *Naglu*^−/−^ mice at ages 3 days, 1 week, 2 weeks, 4 weeks, and 12 weeks were co-stained by IHC for CI-MPR and CD31, and the CI-MPR signal associated with the vasculature in cortical regions was quantified (Fig. 3a, b). At 3 days and 1 week of age, over 80% of cortical vessels evaluated were positive for CI-MPR (84.8 ± 1.2% and 87.7 ± 1.8%, respectively), but by 2 and 4 weeks of age less than 20% of cortical vessels had positive staining for the receptor (17.2 ± 5.2% and 12.7 ± 8.5%, respectively). At 12 weeks of age, less than 1% of cortical vessels were positive for the CI-MPR (0.47 ± 0.30%). These temporal trends were qualitatively observed in cortical regions as well as hippocampus, striatum, and thalamus. Qualitatively, the neuronal CI-MPR signal was similar in *Naglu*^−/−^ and WT mice.

These data demonstrate that the time course of CI-MPR expression on brain vasculature in *Naglu*^−/−^ mice was similar to that of WT mice, suggesting that the developmental regulation of CI-MPR in brain vasculature or neurons is not altered by this disease pathology.

### Systemic treatment with BMN 250 in 12-week-old *Naglu*^−/−^ mice does not lead to normalization of pathology in brain

We next assessed the ability of BMN 250 to cross a potentially diseased and compromised BBB at ages when CI-MPR is not expressed in the brain vasculature of *Naglu*^−/−^ mice. ICV administration of BMN 250 has previously been shown to clear HS and NRE storage, and reverse lysosomal pathology in the brain of *Naglu*^-/-^ mice [1]. Using the same dosing paradigm, four doses of 100 μg (approximately 4 mg/kg) each were administered IV rather than ICV over the course of 2 weeks (i.e., every 3–4 days) in 12-week-old *Naglu*^−/−^ mice. One day after the last dose, brain slices 1 and 3 (Fig. 1) were processed for NAGLU, HS and NRE, and β-hex activity, and brain slices 2 and 4 (Fig. 1) were processed for detection of NAGLU and LAMP2 via IHC.

No positive signal was detected in brains from animals after IV administration using IHC to evaluate regional and cellular NAGLU distribution. NAGLU exposure following IV administration was further evaluated using a 4MU-florescence-based biochemical assay, in which NAGLU activity levels were at or slightly above the assay background level of vehicle-treated *Naglu*^−/−^ mouse brain samples, as shown in Table 1. As also shown in Table 1, this corresponded with ~ 30–40% reductions in HS and NRE, 4–15% reduction in elevated β-hex activity levels, and inconsistent changes in LAMP2 levels across the brain slices. Importantly, none of these five markers are normalized with this dosing paradigm (WT levels have been previously presented [1]).

**Table 1:** Pharmacokinetic and pharmacodynamic endpoints following IV administration of BMN 250 or vehicle to *Naglu*^−/−^ mice

	NAGLU activity (nmol/h/mg protein)	HS (pmol/mg brain)	NRE (pmol/mg brain)	β-hex activity (nmol/h/mg protein)	LAMP2 (no. foci per 4× field)
	Slice 1				Slice 2
Vehicle	2.58 ± 0.39	217.52 ± 10.81	13.59 ± 2.28	5485.24 ± 184.53	194 ± 163
Treated	2.63 ± 0.12	139.65 ± 10.89	8.76 ± 1.42	4636.27 ± 277.75	298 ± 159
*p* value	0.734	9.05E−10	0.000225	4.56E−06	0.217
	Slice 3				Slice 4
Vehicle	1.50 ± 0.19	171.83 ± 34.67	16.11 ± 1.50	4376.51 ± 222.46	668 ± 188
Treated	1.88 ± 0.17	104.79 ± 16.82	11.61 ± 1.72	4202.02 ± 325.74	564 ± 246
*p* value	0.0008	0.000225	6.88E−05	0.231	0.359

Data represent mean ± SD. *p* values = two-tailed unpaired *t* test, *n* = 8 per group, vehicle vs. treated

As noted, β-hex and LAMP2, both markers of lysosomal function, are known to be upregulated more than 2-fold and 3- to 10-fold, respectively, in *Naglu*^−/−^ mouse brain at 12 weeks of age; both are restored to near-normal levels with ICV delivered BMN 250 [1]. Similarly, extensive distribution was observed via IHC when BMN 250 was dosed via the ICV route [1]. These results suggest that BMN 250 does not cross the BBB in therapeutically relevant levels following systemic administration of this dose to *Naglu*^−/−^ mice.

### Increased vascular astrocytosis in *Naglu*^−/−^ mouse brain may pose additional limitations to systemic delivery of therapy to CNS

**Fig. 4:**
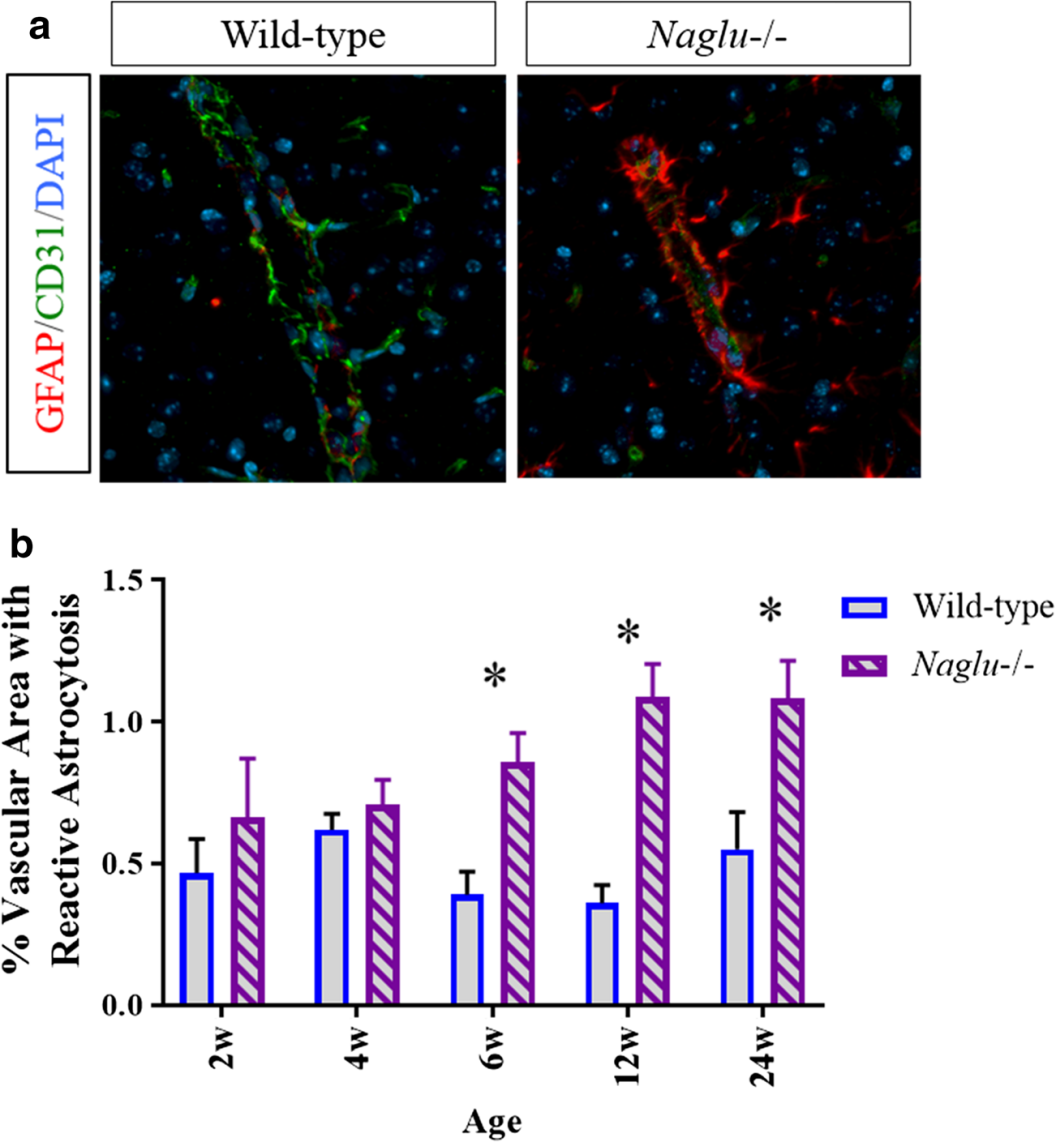
GFAP (red) for astrocytes and CD31 (green) were co-stained (**a**) and the percent of CD31+ vasculature with reactive astrocytosis was quantified at various ages in wild-type and *Naglu*^−/−^ mouse brains. Scale bar = 10 μm (**b**). *p < 0.05, two-way ANOVA with Sidak’s multiple comparisons test, error bars = SEM

Astrocytes play active and passive roles in regulating vascular permeability and BBB integrity [17–19]. Perivascular astrocytosis may form a scar to aid in BBB repair and act as an additional physical barrier in disease states [19, 20]. Vascular astrocytosis was evaluated by analyzing glial fibrillary acidic protein (GFAP) signal surrounding CD31-positive signal. Brains from 2-, 4-, 6-, 12-, and 24-week-old *Naglu*^−/−^ and WT control mice were co-stained by IHC (CD31 and GFAP), and the percent of the vascular area with reactive astrocytosis within 3 μm of CD31 signal was quantified in the cingulate cortex and caudate putamen (Fig. 4a, b). Brain vasculature from 6-, 12-, and 24-week-old *Naglu*^−/−^ mice was associated with significantly more GFAP+ processes than that of WT control mice (6 weeks, WT: 0.390 ± 0.082, *Naglu*^−/−^: 0.858 ± 0.102, *p* = 0.0022; 12 weeks, WT: 0.36 ± 0.0633, *Naglu*^−/−^: 1.086 ± 0.117, *p* = <0.0001; 24 weeks, WT: 0.55 ± 0.131, 24 weeks, *Naglu*^−/−^: 1.082 ± 0.132, *p* = 0.0146).

### CI-MPR is present into adolescence in non-human primate and human brain vasculature

**Fig. 5:**
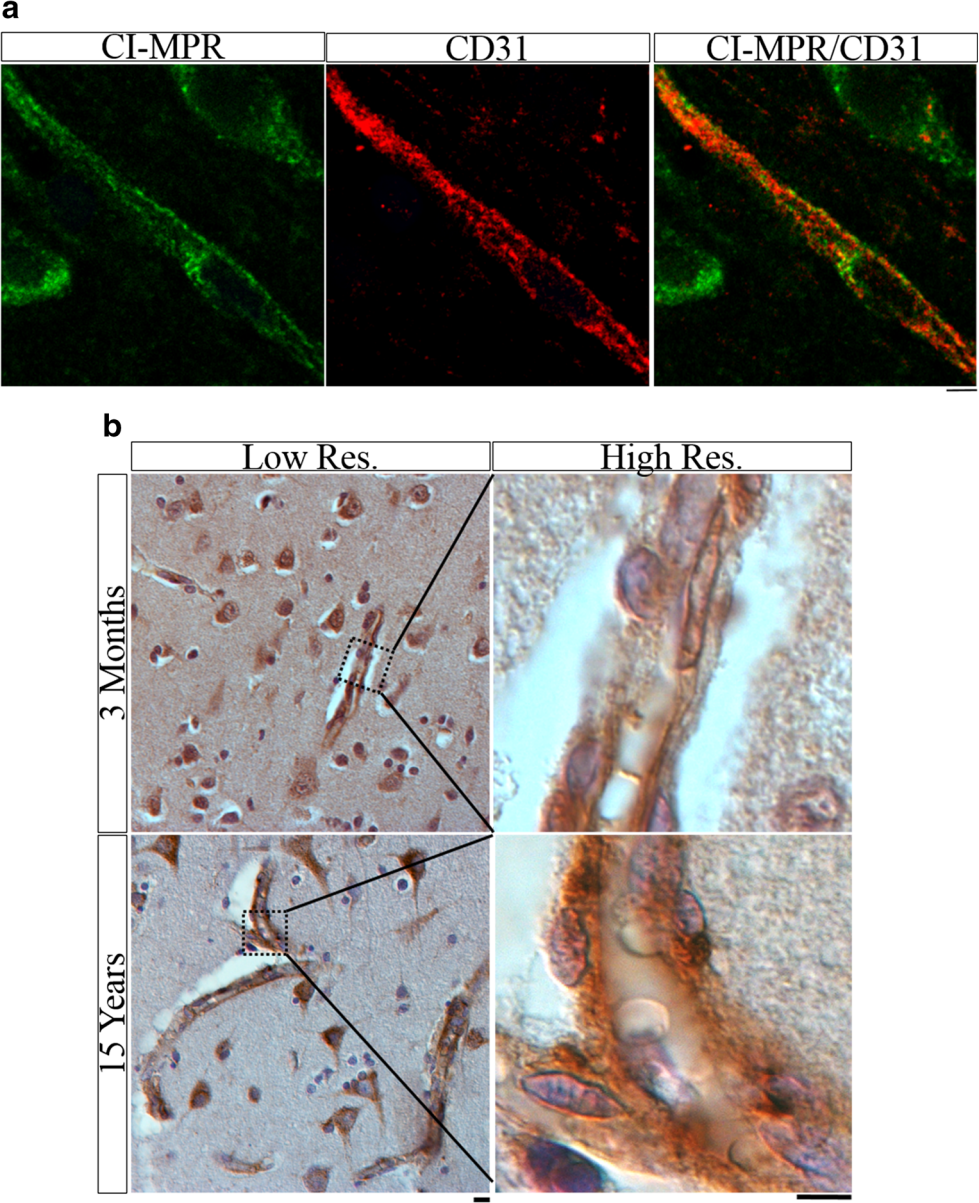
(**a**) Single-slice confocal image of CI-MPR (green) and CD31 (red) co-stain in cortex of healthy 3.9-year-old cynomolgus monkey. Scale bar = 5 μm. (**b**) CI-MPR (brown) IHC in the cortex of normal 3-month-old and 15-year-old human shows vascular and neuronal staining pattern. Low-res. image scale bar = 10 μm. High-res. image scale bar = 5 μm

Bridging from mouse studies, cortical brain tissue was analyzed from two healthy 3.9-year-old (adolescent aged [21]) NHPs (cynomolgus monkeys) and 14 normal human patients whose ages ranged from 2 months (neonate) to 15 years old (adolescent). Co-immunolabeling of the NHP samples for CI-MPR and CD31 demonstrated CI-MPR presence in the brain vasculature on both luminal and abluminal surfaces (Fig. 5a). Because the human tissue was not perfused and contained autofluorescent red blood cells (which interfered with interpretation of immunofluorescence co-staining), chromogenic IHC for CI-MPR was performed and evaluated. Vessels in the human tissue are morphologically distinct, and despite not having a vascular co-stain, there was clear CI-MPR signal in the vasculature at all ages (Fig. 5b).

### Systemic delivery of BMN 250 in juvenile non-human primates does not convey trans-BBB transport

We next assessed the ability of CI-MPR to transport enzyme across the BBB in healthy adolescent NHP, given our results that CI-MPR is still expressed in NHPs at this age. Animals were administered 200 mg/kg BMN 250 via IV administration, or vehicle or 73 mg BMN 250 via ICV administration. Doses selected were the maximum feasible for each route in order to maximize the chance of detecting enzyme in the CNS.

In the CSF, following IV BMN 250 administration, the maximum concentration (*C*_max_) of BMN 250 measured in the CSF of NHPs is 0.3% of the *C*_max_ in plasma (data not shown), suggesting negligible drug passed through the BBB following IV administration. Similarly, following ICV administration, CSF dose-normalized *C*_max_ (DN *C*_max_) is approximately 5000-fold greater than CSF DN *C*_max_ following IV delivery (data not shown).

**Fig. 6:**
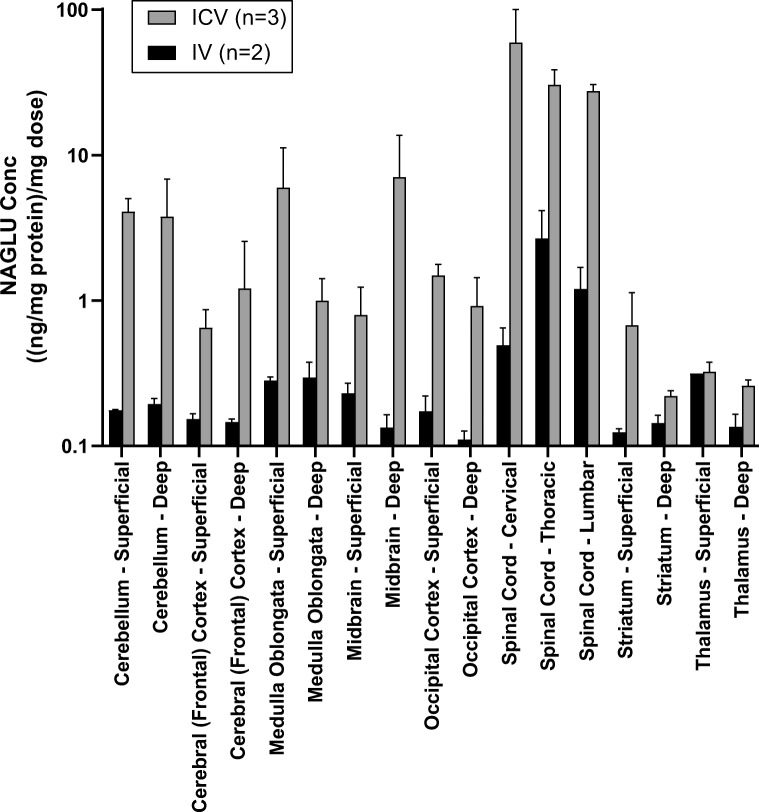
Dose-normalized NAGLU tissue levels show superior biodistribution in cynomolgus monkey following ICV delivery (*n* = 3, light bars) relative to IV delivery (*n* = 2, dark bars) in perfused brain samples collected 48 h after a single dose of BMN 250. Concentrations shown are the measured NAGLU concentration relative to total protein in each sample, normalized to the amount of BMN 250 (mg) dosed. Error bars = SD

In the CNS tissue, ICV administration of BMN 250 in NHPs also resulted in superior biodistribution across deep and superficial (relative to CSF flow) brain and spinal cord regions as compared to IV administration in perfused tissue samples (Fig. 6). Table 2 displays the relative amount of NAGLU across brain regions between IV and ICV drug administration. Overall CNS dose-normalized tissue levels following IV administration were approximately 16.8% of dose-normalized tissue levels following ICV dosing at 48 h following a single dose of BMN 250. Importantly, many brain regions had significantly less distribution (< 5%). As Sanfilippo type B appears to affect the brain broadly [22], distribution to all areas of the brain will be important to treatment. For completeness, absolute ratios of drug distribution to various brain regions are also presented in Table 2. Despite five times higher IV dose, tissue levels of NAGLU in most brain regions are either under or equal to ICV administration.

**Table 2:** Relative CNS tissue levels of NAGLU following IV and ICV administration of BMN 250 to healthy non-human primates

CNS tissue	Dose-normalized % recovery IV/ICV	Absolute % recovery IV/ICV
Cerebellum—deep	5.3%	26.5%
Cerebellum—superficial	4.5%	22.5%
Cerebral (frontal) cortex—deep	16.2%	81%
Cerebral (frontal) cortex—superficial	30.5%	152%
Medulla oblongata—deep	30.5%	152%
Medulla oblongata—superficial	4.3%	21.5%
Midbrain—deep	2.2%	11%
Midbrain—superficial	30.7%	153%
Occipital cortex—deep	14.2%	71%
Occipital cortex—superficial	13.4%	67%
Spinal cord—cervical	0.5%	2.5%
Spinal cord—lumbar	2.5%	12.5%
Spinal cord—thoracic	9.1%	45.5%
Striatum—deep	92.7%	461%
Striatum—superficial	25.6%	128%
Thalamus—deep	55.3%	277%
Thalamus—superficial	62.8%	314%
Mean	23.6%	118%

### NAGLU staining is restricted to the brain vasculature following IV dosing

**Fig. 7:**
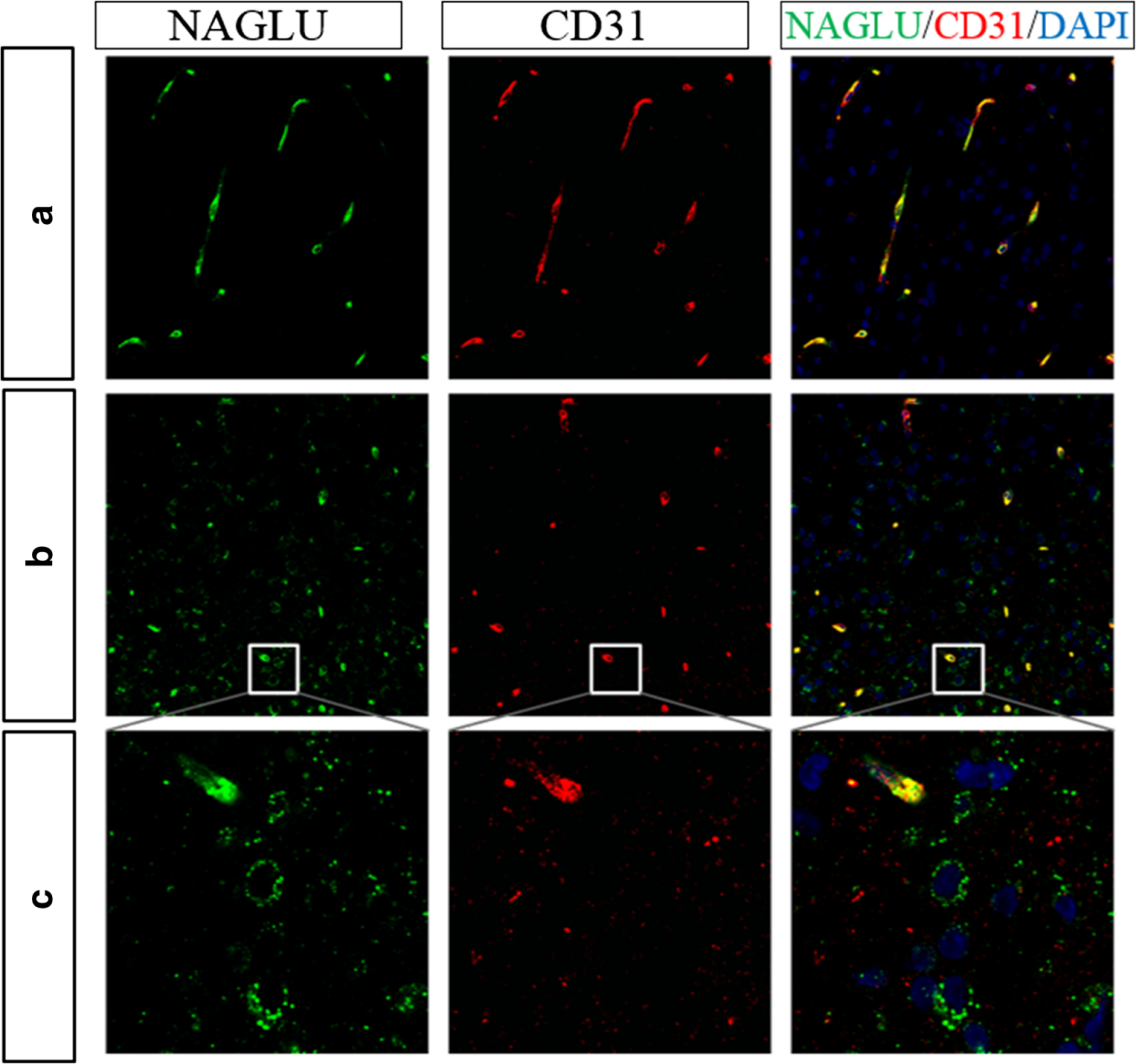
(**a**) Representative staining from superficial medulla oblongata from cynomolgus monkey administered BMN 250 via the IV route. All NAGLU (green) staining is colocalized with CD31 (red) positive endothelial cells, indicating administered NAGLU does not reach target neurons. (**b**, **c**) 20× image with zoom of subfornical organ, showing extravascular NAGLU in a region with less BBB tight junctions. Scale bars = 20 μm

CNS tissue samples from IV BMN 250 administered animals were further analyzed for NAGLU localization via IHC. Select regions with the highest NAGLU concentration following IV administration (superficial medulla oblongata and superficial midbrain) were utilized for IHC to maximize the likelihood of detecting NAGLU across cell types. As shown in Fig. 7a, NAGLU signal is qualitatively restricted to CD31-positive brain vasculature in all regions evaluated, and no NAGLU was detected elsewhere. Previous data has shown WT NAGLU levels are undetectable in untreated animals, presumably due to a lack of cross-reactivity between human and cynomolgus monkey NAGLU or due to limits to assay sensitivity by IHC. Thus, while IV administration of BMN 250 led to detectable levels in sections of brain homogenate, detectable drug appears restricted to vasculature and does not reach brain parenchyma in appreciable amounts. This localization likely explains why relatively more NAGLU was measured in the CNS tissue, which includes the vasculature, than in the CSF following IV dosing in comparison to ICV dosing.

As shown in Figs. 7b and c, some extravascular NAGLU was observed in regions such as the subfornical organ noted for having a reduced BBB. While this amount is qualitatively small, it supports the ability of the NAGLU IHC assay to detect even a limited amount of BMN 250-derived NAGLU protein.

## Discussion

These results demonstrate that in contrast to ICV administration, IV administration of enzyme does not sufficiently distribute to the brain parenchyma in either a mouse model of Sanfilippo type B or in healthy NHPs. To maximize pharmacological benefit, it is believed enzyme delivered to the CNS for the treatment of Sanfilippo type B must be broadly distributed and chronically active in order to be efficacious. In the absence of treatment, the pathological intracellular deposition of HS resulting from NAGLU deficiency leads to global neuronal loss and atrophy, *ex vacuo* dilatation of the lateral ventricles, and white matter abnormalities on brain magnetic resonance imaging [22]. Thus, Sanfilippo type B appears to affect the brain broadly [22]. In *Naglu*^−/−^ mice, elevated brain tissue NAGLU levels following ICV treatment of BMN 250 are associated with increased pharmacological effect, measured as the reduction in total HS and NRE in brain tissue [1]. While clinical presentations were not assessed in the mice, the sustained pharmacological effect is suggestive of the broadly disseminated enzyme activity that is likely required for the treatment of Sanfilippo type B.

This study is the first to directly analyze the post-natal time course of CI-MPR specifically in brain vasculature of WT and *Naglu*^−/−^ mice. These results are in agreement with previous reports of relatively high levels of CI-MPR in the mouse CNS during development followed by post-natal decline that lead to lower levels measured in adulthood [23–29]. Results here identify the age when expression from endothelial cells is reduced and more specifically extend the current understanding of this developmental regulation and add an important distinction: in mice, CI-MPR is regulated differently in endothelial cells than it is in the neurons, as expression is almost absent in adult vasculature but is retained in adult neurons. Importantly, these findings hold in the *Naglu*^−/−^ mouse model, suggesting that the pathophysiology of this disease does not alter the developmental regulation of CI-MPR.

While it has been reported that the BBB of *Naglu*^−/−^ mice is compromised as early as 12 weeks of age potentially allowing ERT to simply “leak” across the BBB [9], the increased vascular astrocytosis in brains of *Naglu*^−/−^ mice shown here may be a compensatory additional barrier to diffusion across the potentially impaired BBB. Increased vascular astrocytosis is consistent with vascular pathology, and significant pathology in the endothelial cells of *Naglu*^−/−^ mouse brains has been reported [9]. Further studies are required to determine the temporal relationship, but it is hypothesized that astrocytosis leads to scarring as a mechanism of sealing the otherwise compromised BBB. Such scarring has been reported to occur after ischemic injury in which there is BBB breakdown [30]. Importantly, the absence of BBB leakage in Sanfilippo type B patients as assessed by magnetic resonance imaging has been reported [31], suggesting that similar barriers to BBB leak may also exist in patients.

In contrast to mice, vascular expression of CI-MPR persists into adolescence in NHP and human CNS tissue. In line with these species differences, CI-MPR is also known to be a sink for excess IGF2 [32], which is developmentally regulated in mouse but not human serum. Specifically, serum levels of IGF2 in rodents have been shown to be high during prenatal periods with a subsequent decline, while IGF2 serum levels do not change substantially between pre- and post-natal time periods in humans [23, 33, 34].

Despite this continued CI-MPR expression at the BBB of juvenile NHPs, dose-normalized CSF concentrations of BMN 250 following ICV administration were approximately 5000-fold greater than those following IV administration after a single dose of BMN 250 in juvenile NHPs. The magnitude of this difference cannot be accounted for by the difference in CSF and blood volumes in cynomolgus monkeys (~ 15 mL [35] and ~ 60–65 mL/kg [36], respectively). Similarly, 0.3% of plasma levels are detected in the CSF at *C*_max_ following IV BMN 250 administration. Further, CNS tissue levels of NAGLU, using an assay specifically for BMN 250 that has been processed through the lysosome (indicating drug reached its target site of action within the cell), demonstrated superior biodistribution following ICV delivery, in contrast to IV, to both superficial and deep CNS tissues relative to the ventricle (Fig. 6). Cumulatively, these data demonstrate the superiority of the ICV route of administration for global BMN 250 CNS biodistribution.

Brains from IV-administered *Naglu*^−/−^ mice showed a modest reduction in HS and NRE substrates in brain tissue (Table 1). Our combined results suggest that this modest effect was likely restricted to the substrate load in or on the luminal side of brain endothelial cells. This predicts a limitation to the pharmacological effect that can be achieved via IV administration. Furthermore, this low level of pharmacological effect did not translate into consistent normalization of downstream markers of lysosomal function, notably LAMP2. Systemically administered mannose 6-phosphorylated NAGLU has also recently been studied in a phase 1/2 clinical trial in patients with Sanfilippo type B [37]. Limited reduction in CSF HS was observed in treated patients, consistent with our findings in *Naglu*^−/−^ mice. These results suggest restriction of IV-administered enzyme to the endothelial cell compartment in mice and NHP and in human patients.

In the work presented here, enzyme levels were not detected specifically in brain parenchyma following IV administration at the maximum feasible dose to healthy NHPs. In contrast, following ICV administration of BMN 250 to *Naglu*^−/−^ mice, NAGLU staining via IHC was observed bilaterally with ~ 70% of NAGLU signal being detected in the neurons. Additionally, ~ 15% was detected in microglia, ~ 10% in astrocytes, and only ~ 5% in the vasculature [1]. In a similar study following intracisternal administration of recombinant human sulfamidase directly into the CSF of SGSH-null dogs, recombinant human sulfamidase was detected via IHC in neurons and glia at 24 h following either: 2 injections of 3 mg each 4 days apart, or 3 weekly injections of 3 mg each [38]. Therefore, it is clear ERT distributes into neurons when delivered directly into the CNS, and CI-MPR facilitates uptake of enzyme to target cell types.

As noted, CI-MPR has been implicated in trans-BBB transport of β-glucoronidase and sulfamidase in a M6P-dependent manner in neonatal but not adult WT mice [7, 8]. Here, we show that in juvenile NHPs, at an age when CI-MPR is expressed by brain vasculature endothelial cells, CI-MPR was not able to transport BMN 250 across the BBB. Importantly, other large animal studies show similar lack of trans-BBB transport with mannose 6-phosphorylated enzyme [39, 40]. This perhaps suggests specific developmental changes to receptor-mediated transport mediated by CI-MPR of mannose 6-phosphorylated enzyme at post-neonatal age despite continued expression, or other developmentally related BBB changes in the mouse.

It is therefore clear that despite CI-MPR expression at BBB endothelial cells in adolescent NHPs and humans, CI-MPR is not capable of transporting enzyme *across* the BBB. It is active, however, in facilitating enzyme uptake to neuronal cells that are exposed to BMN 250 delivered directly to the CSF. Any substrate reduction observed in the CSF or CNS following IV administration of an ERT likely specifically results from substrate clearance in the endothelial compartment, suggesting it will not be possible to normalize pathophysiological endpoints such as substrate, β-hex, or LAMP2, especially to target neuronal cell types, with IV administration of an ERT. Because neurons are the therapeutic target for the treatment of Sanfilippo type B, the demonstration that neuronal expression of CI-MPR is retained in mouse, monkey, and human through development in conjunction with the cell-specific distribution data discussed above (confirming CI-MPR is capable of facilitating uptake) suggest the appropriate biodistribution of BMN 250 for pharmacological activity in patients when dosed via the ICV route. The investigations reported here demonstrate that IV administration of substrates for CI-MPR, such as lysosomal enzymes, does not allow the direct enzyme replacement to target CNS cell types that is required for the treatment of neurological manifestations of lysosomal storage diseases, supporting the value of ICV administration for addressing this significant unmet medical need.

## Conclusions

The treatment of neurological presentations of lysosomal storage diseases remains a significant clinical challenge. BMN 250 is an enzyme replacement therapy in development for the treatment of Sanfilippo type B, a disease with a primarily neurological phenotype. Systemic delivery of BMN 250 did not lead to normalization of pathological symptoms, suggesting that BMN 250 did not significantly cross a potentially compromised BBB in the *Naglu*^−/−^ mouse model. To maximize pharmacological benefit, it is believed that NAGLU must be delivered broadly and directly to the CNS. The results presented here suggest that ICV delivery of BMN 250 throughout the CNS would enhance its therapeutic benefit compared with systemic administration. These findings support the continued investigation of BMN 250 as a potential ICV therapy for Sanfilippo type B.
